# Clinical characteristics and molecular genetic analysis of ten cases of ornithine carbamoyltransferase deficiency in southeastern China

**DOI:** 10.1186/s13052-024-01740-8

**Published:** 2024-09-11

**Authors:** Gaopin Yuan, Zhiyong Liu, Zhixu Chen, Xiaohong Zhang, Weifeng Zhang, Dongmei Chen

**Affiliations:** 1 The Graduate School of Fujian Medical University, Fuzhou, China; 2 Department of Endocrinology, Quanzhou Maternity and Children’s Hospital, Quanzhou, China; 3 Department of Neonatology, Quanzhou Maternity and Children’s Hospital, Quanzhou, China; 4 Department of Intensive Care Medicine, Quanzhou Maternity and Children’s Hospital, Quanzhou, China

**Keywords:** Ornithine transcarbamylase deficiency, Hyperammonemia, Gene mutation, Urea cycle disorders, Acute liver failure

## Abstract

**Background:**

This study aimed to investigate the clinical and molecular genetic characteristics of ten children with ornithine carbamoyltransferase deficiency (OTCD) in southeastern China, as well as the correlation between the genotype and phenotype of OTCD.

**Methods:**

A retrospective analysis was performed on the clinical manifestations, laboratory testing, and genetic test findings of ten children with OTCD admitted between August 2015 and October 2021 at Quanzhou Maternity and Children’s Hospital of Fujian Province in China.

**Results:**

Five boys presented with early-onset symptoms, including poor appetite, drowsiness, groaning, seizures, and liver failure. In contrast, five patients (one boy and four girls) had late-onset gastrointestinal symptoms as the primary clinical manifestation, all presenting with hepatic impairment, and four with hepatic failure.Nine distinct variants of the *OTC* gene were identified, including two novel mutations: c.1033del(p.Y345Tfs*50) and c.167T > A(p.M56K). Of seven patients who died, five had early-onset disease despite active treatment. Three patients survived, and two of them underwent liver transplantation.

**Conclusions:**

The clinical manifestations of OTCD lack specificity. However, elevated blood ammonia levels serve as a crucial diagnostic clue for OTCD. Genetic testing aids in more accurate diagnosis and prognosis assessment by clinicians. In addition, we identified two novel pathogenic variants and expand the mutational spectrum of the gene *OTC*, which may contribute to a better understanding of the clinical and genetic characteristics of OTCD patients.

## Background

Ornithine transcarbamylase deficiency (OTCD; MIM 311250) is an X-linked inborn error of the urea cycle caused by mutations in the gene *OTC* located on chromosome Xp11.4. Consequently, hemizygous males develop this condition. They have a spectrum of severity ranging from complete OTCD, which presents at the neonatal stage with acute hyperammonemia, to partial OTCD, manifesting with mild symptoms and late-onset [1]. The clinical presentation of carrier females also differs greatly, owing to the degree of X-inactivation in hepatocytes [2]. Therefore, OTCD can be easily misdiagnosed.

In the urea cycle, ornithine transcarbamylase (OTC) is required for the production of citrulline and inorganic phosphates from carbamyl phosphate and ornithine. *OTC* variants result in decreased or absent OTC enzyme activity, blocking citrulline synthesis and interrupting the urea cycle. This leads to hyperammonemia and accumulation of orotic acid [3]. The clinical manifestations of hyperammonemia are nonspecific, often resulting in a delay in the diagnosis of OTCD [4]. According to the onset period, OTCD can typically be divided into two groups: early-onset (≤ 30 days) and late-onset (> 30 days). Since the first symptoms of OTCD are extremely variable and molecular genetic testing is necessary for diagnosis, it is essential to accumulate information on genetic analyses and clinical phenotypes to determine genotype-phenotype correlations. This study reports the clinical and biochemical features and molecular characteristics of ten Chinese patients with OTCD. We also reviewed the relevant literature and analyzed the relationship between the phenotypes and genotypes.

## Materials and methods

### Study subjects

Ten patients diagnosed with OTCD were recruited between August 2015 and October 2021 at Quanzhou Maternity and Children’s Hospital of Fujian Province in China.Diagnosis of OTCD was based on clinical features, specific biochemical criteria, and molecular analyses. A retrospective review of clinical manifestations and laboratory tests, including blood ammonia levels, liver function tests, tandem mass spectrometry (MS/MS), urine organic acids, in addition to color ultrasound, was conducted. This study was approved by the Ethics Committee of the Quanzhou Maternity and Children’s Hospital, China. The patients’ parents provided written informed consent for participation in the study.

### Genetic analysis

After obtaining informed consent, 2 mL of peripheral blood was collected from patients and their parents. Whole-exome sequencing (WES) was performed on samples from the ten patients. Raw reads were processed by fastp, followed by paired-end sequencing of reads aligned to the human GRCh37/hg19 genome using the Burrows-Wheeler Aligner (BWA) software package. GATK software (https://software.Broadinstitute.org/gatk/) was used for SNPs and indel calling. Mutations were described according to the HGVS recommendations (http://wwwhgvs.org/mutnomen/recsprot.htm1). PolyPhen-2, PROVEAN, Mutation Taster, and Sorting Intolerant From Tolerant (SIFT) were used to analyze the potential pathogenicity of the missense mutations. Amino acid conservation was predicted using phyloP and phastCons, and homologous amino acid sequences were compared using DNAMAN software. The pathogenicity of all variants was evaluated according to the American College of Medical Genetics and Genomics (ACMG) clinical practice guidelines [5]. These variants were considered novel if they were not found in the Human Gene Mutation, ClinVarMiner, or Exome Aggregation databases. The results were verified using Sanger sequencing.

## Results

### Clinical features of OTCD patients

Table 1 presents the demographic and clinical findings of ten patients with OTCD from nine unrelated families. All patients appeared normal at birth. Five patients (one boy and four girls) had a late-onset form of the disease, with a median age of 3.2 years (ranging 1.5 years to 6.3 years) and normal growth and development until the time of the first clinical manifestations. The most prevalent symptoms observed in these five patients were gastrointestinal distress, including vomiting and feeding refusal, followed by liver dysfunction and psychiatric symptoms. Treatment was initiated with a low-protein diet, intravenous fluid and energy replenishment, arginine supplementation to reduce blood ammonia, continuous renal replacement therapy (CRRT). Two died of hyperammonemia. Three exhibited clinical remission, and two of them underwent liver transplantation with normal growth and development after surgery. The other girl experienced multiple hospitalizations due to hepatic dysfunction and hyperammonemia, and she also experienced mild intellectual disability. Five male patients presented with early-onset symptoms within 1–7 days after birth, including poor appetite, drowsiness, groaning, and seizures. Initially misdiagnosed as neonatal infection or neonatal encephalopathy, the patients’ condition progressed to seizures and coma within 1–2 days, resulting in death within 10 days after birth.

**Table 1:** Clinical features and laboratory findings at presentation in 10 patients with OTCD

patient No	Sex	Onset age	Type	Presenting features	AMON(10–47µmol/L)	Peak AMON (µmol/L)	ALT(0-40U/L)	AST(0-40U/L)	CIT(5–35 umol/L)	Uracil(0-7mmol/molCr)	Urine orotic acid(0-2.5 mmol/molCr)	Color doppler ultrasound of liver	Family history
1	F	2.7y	LO	Repeated vomiting, poor appetite, liver dysfunction	468	483	647	126	15.1	8.1	0	Enhanced echo of liver arenchyma	NO
2	F	3.5y	LO	Psychiatric symptoms, Vomiting, lethargy	450	753	276	73	12.74	142.04	1407.81	Enhanced echo of liver arenchyma	NO
3	M	6.3y	LO	Abdominal pain, Vomiting, lethargy	101	135	129	154	3.39	75.5	321.8	normal	NO
4	F	1.5y	LO	Vomiting, liver dysfunction	248	428	267	184	19.46	95.33	35.54	normal	Yes, an older brother died of unknown cause 3 days after birth
5	M	1d	EO	drowsiness, poor appetite	280	725	32	50	2.55	ND	ND	ND	Yes, Is the younger brother of P4
6	M	2d	EO	drowsiness, groan	1011	1020	48	42	4.95	0	34.65	ND	NO
7	F	1.8y	LO	Abdominal distention, Vomiting, poor appetite,	150	222	4670	2135	2.9	65.3	39.7	Enhanced echo of liver arenchyma	NO
8	M	1d	EO	drowsiness, groan, poor appetite	360	630	17	68	3.85	12.87	470.66	ND	Yes, His maternal grandmother gave birth to 8 children, 4 daughters were alive and 4 sons died 2–3 days after birth.
9	M	2d	EO	drowsiness, poor appetite, tachypnea, Seizures	400	400	19	78	2.66	4.68	132.92	ND	NO
10	M	7d	EO	jaundice, poor appetite, Seizures	3620	3620	578	416	5.88	5.11	99.56	ND	NO

M, male; F, female; d, days; y, years; EO, early onset; LO, late onset; CIT, blood citrulline; AMON, blood ammonia; ND, not detected

### Biochemical results and liver ultrasound at OTCD diagnosis

Table 1 summarizes the results of the biochemical analyses for each patient. Notably, elevated blood ammonia levels were observed in all patients tested. Peak ammonia levels were significantly higher in the early-onset group than those in the late-onset group, indicating a potential association with disease severity. In the late-onset group, all patients exhibited elevated transaminase levels. Four of them (P1, P2, P4, and P7) experienced liver failure, while only one patient in the early-onset group did. Urinary orotic acid levels increased in all patients tested except for one, for whom specimens were retained after CRRT therapy. Only six patients demonstrated decreased serum levels of citrulline. Five patients underwent color Doppler ultrasound examination of the liver, and three showed hyperechoic livers.

### Mutation analysis of the *OTC* gene

**Fig. 1:**
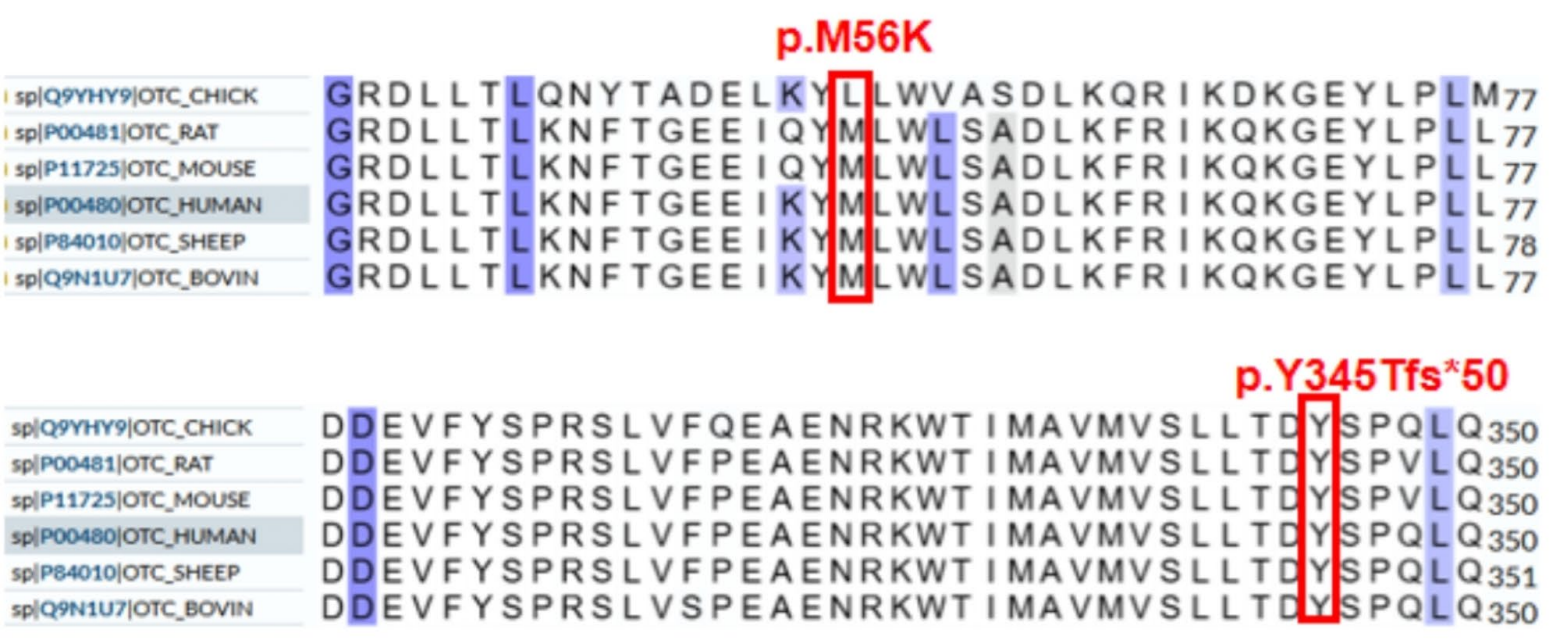
A conservative analysis of the two new loci

We detected nine distinct mutations in the *OTC* gene in ten patients, consisting of six missense mutations, two frameshift mutations, and one gross deletion (Table 2). Two of these mutations were previously unreported (NM_000531.6): c.1033del and c.167T > A. According to the ACMG guidelines, the variant c.1033del (p.Y345Tfs*50) was evaluated as pathogenic (PVS1 + PS2_Moderate + PM2) and c.167T > A (p.M56K) as likely pathogenic (PM1 + PM2 + PM5 + PP3). Multiple sequence alignment using Clustal X was performed. The methionine residue at position 56 and tyrosine residue at position 345 (highlighted by a red box) are highly conserved among different species (Fig. 1). The remaining seven variants (c.140del, c.1028 C > G, c.116G > T, c.674 C > T, exon5 del, c.77G > A, and c.725 C > T) have been documented in Human Gene Mutation Database(HGMD), ClinVar, and the literature. Both variants identified in P1 and P2 were de novo and were not detected in the peripheral blood of their mother. The mutations in the remaining eight patients were inherited from their healthy mothers.

**Table 2:** Molecular results and clinical outcomes identified in 10 patients with OTCD

Patient No	Chromosome position	Exon	Nucleotide change	Amino acid change	Variation type	Nature of mutation	Clinical type	Outcome
1	ChrX:38,280,302	E10	c.1033del	p.Y345Tfs*50	frameshift variation	De novo	LO	Liver Transplantation, Alive
2	chrX:38,226,606	E2	c.140del	p.N47Tfs*17	frameshift variation	De novo	LO	Deceased
3	chrX:38,280,298	E10	c.1028 C > G	p.T343R	missense variation	Inherited	LO	Liver Transplantation, Alive
4	chrX:38,226,582	E2	c.116G > T	p.G39V	missense variation	Inherited	LO	Deceased
5	chrX:38,226,582	E2	c.116G > T	p.G39V	missense variation	Inherited	EO	Deceased
6	chrX:38,226,633	E2	c.167T > A	p.M56K	missense variation	Inherited	EO	Deceased
7	chrX38268005	E7	c.674 C > T	p.P225L	missense mutations	Inherited	LO	low-protein diet, Drug
8	chrX:38,401,275–38,401,428	E5	exon5 del NM_000531.6	ND	CNV	Inherited	EO	Deceased
9	chrX:38,212,026	E1	c.77G > A	p.R26Q	missense variation	Inherited	EO	Deceased
10	chrX:38,268,136	E1	c.725 C > T	p.T242I	missense variation	Inherited	EO	Deceased

## Discussion

The clinical symptoms of OTCD are non-specific and often misdiagnosed as gastrointestinal disease or liver disease, food or drug poisoning, or central nervous system infection. This can delay diagnosis and treatment, leading to a high mortality rate [6–8]. Hyper-ammonemia is a critical diagnostic indicator. In this study, five male patients presented with early-onset symptoms. Clinical manifestations of affected subjects include eating difficulties, drowsiness, groaning, and seizures. The main symptoms in our late-onset patients were poor appetite, vomiting, lethargy and abnormal liver function.

Blood ammonia levels should be promptly tested in patients with unexplained gastrointestinal symptoms, abnormal liver function, or encephalopathy. Early-onset patients exhibited significantly increased ammonia levels, while late-onset ones exhibited greater variability. Among our late-onset patients, two with significantly increased blood ammonia levels died despite active treatment, while two children who underwent liver transplantation survived. One patient (P7) with moderately increased blood ammonia levels improved after receiving low protein diet and arginine treatment, but later presented with recurrent hyperammonemia and mild intellectual disability. These cases underscore the high mortality rate associated with OTCD and highlight the crucial role of blood ammonia levels in determining the disease severity and prognosis.

The correlation between OTCD and liver complications is increasingly recognized, with over 50% of symptomatic OTCD patients experiencing such condition [9–12]. In some instances, acute liver diseases(ALF) may be the first sign of OTCD [10, 12]. Furthermore, patients with acute hyperammonemia may experience recurrent episodes of ALF [11, 13]. ALF is the most common complication in male newborns with severe OTCD [10, 11]. In this study, all four girls with late-onset OTCD had ALF, whereas only one of five boys with early-onset OTCD experienced ALF. More recently, a comprehensive review has summarized that acute liver failure is a more frequent observation in OTCD, but the specific underlying mechanisms are still not well elucidated [9]. Moreover, a late-onset patient with a peak blood ammonia level of 222 mol/L developed severe ALF, indicating that the blood ammonia level is not always the direct cause of liver failure. Studies have proposed that the inhibition of liver protein synthesis rather than nonspecific cell death due to hyperammonemia is a potential mechanism in hyperammonemia-induced ALF [11]. One study showed that patients had a high rate of liver hyperechogenicity, and more precisely in the 53% of female patients and 42% of male ones [9]. In our study, five patients underwent color Doppler ultrasound examination of the liver, of which three showed a hyperechoic liver and one did not. The specific significance of liver hyperechogenicity is unclear.Further research is needed to investigate the underlying mechanisms of liver disease in OTCD to provide better treatment strategies for this complication.

The interaction between genomic and epigenetic factors leads to different phenotypes of disease [14],*OTC* mutations show high heterogeneity [1, 6]. In this study, nine *OTC* gene variants were detected in ten patients: seven were previously reported, while two variants, c.1033del and c.167T > A are novel. The heterozygous deletion of c.1033del resulted in a frameshift mutation, leading to the formation of a truncated protein (p.Y345Tfs*50) and a loss-of-function variant, identified by ACMG as pathogenic (PVS1 + PS2_Moderate + PM2). The c.167T > A missense variant is located in a highly conserved region of OTC enzymes at the junction of three crucial helices (helices 1, 5, and 11) that bridge the two domains of the protein [15]. Therefore, this variant may affect interdomain alignment, thus affecting enzyme activity, as well as a mutation in the pathogenic amino acid residue p.M56T at this locus, which has been reported to be related to late-onset OTCD [15–17]. According to the pathogenicity classification of ACMG, this is likely to be a pathogenic mutation (PM1 + PM2 + PM5 + PP3).

Disease severity depends on OTC activity, which is influenced by the type and site of mutations [16, 18] and environmental factors, including daily protein intake [7, 8]. The complete loss of OTC function caused by large deletions, frameshifts, or nonsense mutations often leads to a severe neonatal onset [16, 17]. This study found P8 with the deletion of exon 5 of the *OTC* gene exhibiting early-onset, while two patients (P1 and P2) with frameshift mutations who showed severe late-onset. The c.140del variant of P2 has also been reported in a Chinese female patient who died after drug treatment [6].

Some missense mutations can cause a complete loss of OTC function, leading to severe neonatal-onset diseases in hemizygous males and most symptomatic heterozygous females. Missense mutations which retain some OTC activity can cause late-onset in hemizygous males [16, 17]. In this study, we detected the missense mutation c.1028 C > G in P3, which has a relatively mild late-onset clinical presentation. This mutation was previously found in a male late-onset patient without neurological damage [19], suggesting that it may partially preserve OTC activity and present as late-onset in male subjects. P225L is a relatively common mutation in the Chinese OTCD population [6]. It has been identified in several male patients with early-onset, all of whom experienced severe hyperammonemia and died during the neonatal period [20–24]. This indicates that the P225L variant is associated with severe male early-onset. Although the correlation between genotype and phenotype is not yet clear, genetic testing can support the clinician in providing a more precise diagnostic evaluation and solid evidence for counselling to the affected families [25], and it should facilitate the functional study on the proteins in urea cycle.

In this study, among the seven patients with parental validation, five had mutations in healthy mothers, including two female patients with severe hyperammonemia whose mothers were also healthy. Members of the same family with the same mutation can have great differences in phenotype. Moreover, the clinical manifestations of OTCD in affected women within the same family may differ [20, 26] due to random X chromosome inactivation or mosaicism, epigenetics, diet, lifestyle, nutrition, and other factors [2, 27]. This highlights the diversity of clinical manifestations of late-onset OTCD and the multi-factor influence of the *OTC* gene, which further complicates the prenatal diagnosis of female heterozygotes.

## Conclusions

Overall, we analyzed the clinical and genetic characteristics of ten patients with OTCD and identified two novel *OTC* pathogenic variants. The clinical symptoms of the disease are nonspecific. However, hyperammonemia is an important diagnostic clue and is related to prognosis. Genetic testing not only helps to make a definitive diagnosis, but also supports the clinician in providing a more precise prognostic evaluation. More samples are needed to deeply explore the correlation between genotype and phenotype. The prognosis of OTCD is closely related to liver complications. Further studies are needed to better understand the potential mechanisms.

## Data Availability

The datasets used and analyzed in the current study are available from the corresponding author upon reasonable request.

## Abbreviations

OTCD: Ornithine carbamoyltransferase deficiency
OTC: Ornithine transcarbamylase
MS/MS: Tandem mass spectrometry
WES: Whole-exome sequencing
ACMG: American Academy of Medical Genetics and Genomics
UCD: Urea cycle disorder
ALF: Acute liver diseases
CRRT: Continuous renal replacement therapy
